# Supplementary material for: WordCommentsAnalyzer: A windows software tool for qualitative research
Source: F1000Res. 2018 Sep 4;7:536. Originally published 2018 May 3. [Version 2] doi: 10.12688/f1000research.14819.2 (PMC6137411; doi:10.12688/f1000research.14819.2)
Supplement: Supplementary file 3 [file f1000research-7-17614-s0002.tgz › b2a79496-4ada-47c4-9682-a99ce867836d.zip!/Eight Iranian Health Journals/Yafteh _14.docx]

© Yafteh  . This work is licensed under CC BY 4.0 (https://creativecommons.org/licenses/by/4.0/).

Volume & Issue: دوره 16 - شماره 4

Title: امکان‌سنجی اجرای پروژه یادگیری الکترونیک در دانشگاه علوم پزشکی لرستان از دیدگاه اساتید، دانشجویان، مدیران و کارکنان اجرایی در سال تحصیلی 92-91

http://yafte.lums.ac.ir/article-1-1787-fa.html

مقدمه: امروزه یادگیری الکترونیک به عنوان یکی از بسترهای مؤثر آموزشی محسوب می‌شود و با توجه به قصد دانشگاه علوم پزشکی لرستان برای پیاده‌سازی این نوع آموزش، پژوهش حاضر به بررسی امکان اجرای یادگیری الکترونیک در دانشگاه از دیدگاه اساتید، دانشجویان، مدیران و کارکنان اجرایی در سال تحصیلی 92-91 پرداخته است. بحث و نتیجه‌گیری: در حال حاضر آمادگی لازم برای اجرای پروژه یادگیری الکترونیک در دانشگاه وجود ندارد و برای پیاده‌سازی این شیوه آموزشی باید زیرساخت‌های مربوط فراهم گردد. یافته‌ها: دانشگاه علوم پزشکی لرستان از نظر آمادگی تجربه آموزشی و فنی، آمادگی فرهنگی، آمادگی تجهیزات، آمادگی شبکه ارتباطی، آمادگی نیروی انسانی، آمادگی مدیریت، آمادگی سیاست‌های آموزشی، آمادگی امنیت و آمادگی پشتیبانی در سطح مطلوبی قرار ندارد و فقط از نظر قوانین و مقررات در سطح مطلوبی قرار دارد. مواد و روش‌ها: روش مطالعه، توصیفی-مقطعی بوده است. تعداد 316 نفر دانشجو، 84 نفر عضو هیئت علمی و سیزده نفر از مدیران و کارکنان به روش سرشماری انتخاب شدند. برای جمع‌آوری داده‌ها از پرسش‌نامه چند بعدی داراب (آمادگی تجربه آموزشی و فنی، آمادگی فرهنگی، آمادگی تجهیزات، آمادگی شبکه ارتباطی، آمادگی قوانین و مقررات، آمادگی منابع انسانی، آمادگی مدیریت، آمادگی سیاست‌های آموزشی، آمادگی امنیت، آمادگی پشتیبانی) استفاده شد. روایی و پایایی ابزار مورد استفاده با روایی محتوایی و فرمول آلفای کرونباخ (82/0) تأیید شد.

Title: ارزیابی سطوح سرمی مالون دی آلدئید و ظرفیت تام آنتی اکسیدانی پس از مصرف آترواستاتین در بیماران مبتلا به گرفتگی عروق کرونر

http://yafte.lums.ac.ir/article-1-1789-fa.html

مقدمه: استرس اکسیداتیو در پاتوژنز بسیاری از بیماریها دخالت دارد. شرایط استرس اکسیداتیو و عدم تعادل آنتی اکسیدان نقش عمده ای در بروز و توسعه بیماری آترواسکلروز دارد. هدف این مطالعه ارزیابی سطوح سرمی مالون دی آلدئید (MDA) بعنوان شاخص پراکسیداسیون لیپیدی و ظرفیت تام آنتی اکسیدانی (TAC) پس از مصرف آترواستاتین در بیماران مبتلا به گرفتگی عروق کرونر است. بحث و نتیجه‌گیری: در نهایت یافته ها اشاره دارد که یک کاهش معنی دار سطوح سرمی MDA بعلاوه یک افزایش معنی‌دار TCAدر بیماران مبتلا به گرفتگی عروق کرونر با افزایش مدت زمان دریافت آترواستاتین در کاهش دادن استرس اکسیداتیو این بیماران همراه می باشد. یافته‌ها: غلظت سرمی TAC در بیماران با دریافت بیش از 90 روز آترواستاتین mmol/l 101/0±830/0 در قیاس با گروه بیماران با دریافت کمتر از 6 روز mmol/l 076/0±686/0 بطور معنی داری افزایش دارد (001/0=P) درحالیکه سطوح MDA در این بیماران بترتیب µmol/l 112/0±472/0 و µmol/l43/0±73/0 بطور معنی دار کاهش می یابد (029/0P=). مواد و روش‌ها: این مطالعه در 75 بیمار مبتلا به گرفتگی عروق کرونر تأیید شده با آنژیوگرافی انجام شد. بیماران بر اساس مدت زمان مصرف آترواستاتین با دوز mg/day20 به سه گروه تقسیم شدند: 25 بیمار با مصرف کمتر از 6 روز، 27 بیمار با مصرف 6 تا 90 روز و 23 بیمار با مصرف بیش از 90 روز. اندازه گیری سطوح سرمی M‏DA و TCA بترتیب با روش تیوباربیتوریک اسید و بنزی انجام پذیرفت.

Title: بررسی ارزش سیستم نمره دهی CRIB II در پیش بینی مرگ نوزادان نارس

http://yafte.lums.ac.ir/article-1-1790-fa.html

مقدمه: میزان زنده ماندن نوزادان نارس بستگی به سن داخل رحمی، وزن هنگام تولد و وضعیت آنها در هنگام بستری شدن در بیمارستان دارد. سیستم های نمره دهی مختلفی در جهت پیش بینی میزان مرگ و میر در نوزادان طراحی شده است. هدف از این مطالعه نیز بررسی ارزش سیستم نمره دهی CRIB II در پیش بینی مرگ نوزادان با وزن تولد کمتر از 1500 گرم می باشد. مواد و روش‌ها: در این مطالعه توصیفی– تحلیلی که بمدت 8 ماه از شهریور ماه 1389 تا فروردین ماه 1390 انجام شد، نوزادان نارس بستری شده در بخش مراقبت های ویژه بیمارستان امام رضا (ع) کرمانشاه که وزن تولد کمتر از 1500 گرم و سن داخل رحمی کمتر از 32 هفته داشتند و درطی 12 ساعت اول بعد از تولد بستری شده بودند بر اساس سیستم نمره دهی CRIB II، مورد ارزیابی قرار گرفتند. بحث و نتیجه‌گیری: مطالعه ما نشان داد که شاخص CRIB II دارای ارزش بالایی در پیش بینی مرگ نوزادان با وزن تولد کمتر از 1500 گرم می باشد یافته‌ها: از 136 نوزاد مورد مطالعه، 50 نوزاد (8/36%) زنده ماند و 86 نوزاد (2/63%) فوت نمود. میانگین نمره CRIB IIدر نوزادان زنده مانده 9/2±8/5 و در نوزادان فوت شده 9/2±8/9 بود (001/0< P). بر اساس AUC نمره CRIB IIتوانست در حدود 85% (92-77: CI)موارد مرگ را پیش بینی نماید. همچنین بر اساس منحنی ROC بهترین نقطه برش برای نمره CRIB II 5/6 تعیین گردید.

Title: اثرات سلنیت سدیم بر نیم رخ چربی سرم و شاخص های آتروژنی سرم در موش های صحرایی نر دیابتیک

http://yafte.lums.ac.ir/article-1-1791-fa.html

مقدمه: شیوع دیابت درجهان درحال افزایش است. هدف این تحقیق بررسی اثرات حمایتی سلنیت سدیم بر چربی هایی سرم و شاخص های آتروژنی سرم در موش های صحرایی نر دیابتی شده نوع یک تحت اثر آلوکسان است. بحث و نتیجه‌گیری: نتایج بدست آمده از این مطالعه نشان داد که سلنیت سدیم اثرات مفیدی بر قند خون و میزان چربی هایی سرم و شاخص های آتروژنی موش های صحرایی دیابتی نوع یک دارد. یافته‌ها: سلنیت سدیم باعث کاهش معنی دار میزان گلوکز خون، تری گلیسرید، کلسترول، LDL، VLDL، و شاخص های آتروژنی سرم در گروه دیابتی درمان شده با سلنیت سدیم شد. میزان HDL در گروه درمان شده به طور معنی داری افزایش یافت. مواد و روش‌ها: به طور تصادفی سی عدد موش صحرایی نر نژاد اسپراگ به سه گروه تقسیم شدند. گروه اول کنترل، گروه دوم دیابتی درمان نشده و گروه سوم دیابتی درمان شده با سلنیت سدیم با دوز(mg/kg 1 )گروه های دوم و سوم از طریق تزریق تتراهیدرات آلوکسان (mg/kg 120) دیابتی شدند. بعد از هشت هفته درمان حیوانات را بی هوش کرده و از آنها خون تهیه شد و میزان گلوکز خون، تری گلیسرید، کلسترول، لیپوپروتئین با چگالی کم (LDL)، لیپوپروتئین با چگالی خیلی کم (VLDL)، لیپوپروتئین با چگالی بالا (HDL) و شاخص های آتروژنی سرم اندازه گیری شد.

Title: مقایسه سرانجام زایمان با اکسی توسین و خودبخودی در زنان باردارکم خطر

http://yafte.lums.ac.ir/article-1-1792-fa.html

مقدمه: درسال های اخیر، مداخلات پزشکی در لیبر شایع شده است که استفاده از اکسی توسین برای تسریع در زایمان یکی ازآنها می‌باشد. متأسفانه تجویز بی رویه آن می تواند منجر به عوارض مادری و نوزادی شود. هدف این مطالعه، مقایسه سرانجام زایمان با اکسی توسین و خودبخودی در زنان باردارکم خطرمی باشد. بحث و نتیجه‌گیری: نتایج مطالعه حاضر نشان داد که سرعت بخشیدن به روند زایمان یک روش امن نیست و میزان عوارض مادری و نوزادی را افزایش می دهد. بنابراین، در صورت وجود دلایل پزشکی می توان از آن استفاده کرد. یافته‌ها: یافته ها نشان داد اکسی توسین به طور قابل ملاحظه ای باعث افزایش میزان اپی زیاتومی، پارگی کانال زایمان، خونریزی بعد از زایمان، بستری نوزاد در بخش مراقبت های ویژه و کاهش نمره آپگار دقیقه اول و پنجم نوزاد شد که از نظر آماری ارتباط معنی دار بود (05/0 ˂P). مواد و روش‌ها: در این مطالعه توصیفی- مقایسه ای، 395 زن با جنین تک قل، نمایش سر، زایمان طبیعی ، درمرحله فعال زایمان وارد مطالعه شدند. 197 نمونه باتوجه به دستور پزشک اکسی توسین دریافت کردندکه درگروه زایمان با اکسی توسین و 198 نمونه بدون دریافت اکسی توسین درگروه زایمان خودبخودی قرارگرفتند. در دو گروه ، برخی از پیامدهای مادری و نوزادی مانند عوارض مادری، نمره آپگار دقیقه اول و پنجم ، ورود به بخش مراقبت های ویژه نوزادان و... بررسی شد.داده‌ها با استفاده از نرم افزار 15 SPSS و آزمون‌های تی، کای دو و من ویتنی مورد تجزیه و تحلیل قرار گرفت.

Title: ارزیابی ترکیبات آلی فرار در هوای شهر خرم آباد و مقایسه آن با استانداردهای موجود

http://yafte.lums.ac.ir/article-1-1793-fa.html

مقدمه: اولین گام در راستای کنترل آلاینده ها، اندازه گیری و تجزیه آنهاست، زیرا بدون آگاهی کامل ازکیفیت و کمیت آلاینده ها امکان مقایسه با استاندارد های مربوطه و نهایتاً کنترل آنها عملی نخواهد بود. مطالعه حاضر با هدف ارزیابی و تعیین مقدار ترکیبات آلی فرار در هوای شهر خرم آباد انجام شد. مواد و روش‌ها: این پژوهش یک مطالعه توصیفی می‌باشد. در کل از هوای شهر خرم آباد 144 نمونه هوا بطور تصادفی در طول سال گرفته شد. نمونه برداری با استفاده پمپ نمونه بردار محیطی و لوله های جاذب کربن فعال صورت گرفت. روش نمونه برداری بصورت تصادفی- مداوم در طول 24 ساعت شبانه روز بوده است. آماده سازی نمونه و استخراج آلاینده ها به وسیله حلال دی سولفید کربن انجام گرفت و تجزیه نمونه ها توسط دستگاه گاز کروماتوگرافی طیف سنج جرمی مجهز به ستون های مویی انجام گردید. تحلیل داده ها به کمک نرم افزار SPSS-17و استفاده از آزمون آماری T انجام شد. بحث و نتیجه‌گیری: با توجه به نتایج این پژوهش مبنی بر هفت برابر بودن غلظت ترکیبات آلی فرار در هوای شهر خرم آباد نسبت به استاندارد های جهانی، می توان نتیجه گرفت که پیشگیری از آلودگی هوا برای شهر خرم آباد ضروری است و نیازمند طراحی یک سیستم مدیریت مناسب، برنامه ریزی صحیح، سیستم پایش مداوم، و ارتقای سطح آگاهی مردم می باشد. یافته‌ها: نتایج پژوهش حاضر نشان داد که مجموع غلظت ترکیبات آلی فرار در هوای شهر خرم آباد µg/m3 9/1140 بوده که حدود 7 برابر بیشتر از حداکثر غلظت مجاز USEPA می باشد. همچنین یافته های تحقیق نشان داد که بطور کلی میانگین غلظت کلیه BTEX در نقاط مختلف شهر خرم آباد بیشتر از حد مجاز استاندارد EPA برای هوای تنفسی است. در مقایسه میانگین غلظت ترکیبات آلی فرار در هوای شهر خرم آباد در فصول گرم و سرد سال، نتایج آزمون آماری T نشان داد که بین غلظت بنزن و تولوئن و فصل سال رابطه معنی داری وجود دارد (05/0P<). اما در خصوص سایر هیدرو کربن های شناسایی شده با وجود بیشتر بودن غلظت آنها در فصول گرم، رابطه معنی داری وجود نداشت.

Title: بررسی اثرات ضد دردی گیاه کاکوتی (Ziziphora tenuior L.) در موش کوچک آزمایشگاهی نر بالغ

http://yafte.lums.ac.ir/article-1-1794-fa.html

مقدمه: کاکوتی یکی از گیاهان دارویی سنتی است. از ترکیبات شیمیایی فعال و اصلی این گیاه ماده ای تحت عنوان Pulegone می‌باشد که اثرات ضد دردی و ضد التهابی آن به خوبی مشخص شده است. لذا هدف از پژوهش حاضر بررسی اثرات ضد دردی گیاه کاکوتی (Ziziphora tenuior L.) بومی منطقه سیرجان (پاریز) در موش آزمایشگاهی نر بالغ است. بحث و نتیجه‌گیری: داده های تحقیق نشان داد که گیاه دارویی کاکوتی، اثرات ضد دردی بر موش های کوچک آزمایشگاهی دارد و گیاه مذکور بایستی در درمان های آینده مورد توجه قرار گیرد و جایگزین مناسبی برای داروهای شیمیایی می باشد یافته‌ها: نتایج پژوهش حاضر نشان داد، تزریق درون صفاقی گیاه کاکوتی منطقه سیرجان (پاریز) در غلظت های 50، 100 و 150 میلی گرم بر کیلوگرم وزن بدن همانند مورفین موجب کاهش فاز اولیه و ثانویه درد القا شده توسط فرمالین در موش نر بالغ می شود. مواد و روش‌ها: در این پژوهش، پس از جمع آوری برگ و سرشاخه گیاه کاکوتی از منطقه سیرجان عصاره گیری به روش خیساندن صورت پذیرفت. فعالیت ضد دردی با استفاده از تست فرمالین مورد ارزیابی قرار گرفت. از 6 گروه موش های در نظر گرفته شده به 3 گروه، عصاره هیدروالکلی در غلظت های 50، 100 و 150 میلی گرم بر کیلوگرم وزن بدن به صورت درون صفاقی تزریق شد. گروه کنترل بدون درمان و گروه شاهد حلال دارو یعنی سرم فیزیولوژی را به صورت داخل صفاقی دریافت نمود. گروه نیز مورفین را به میزان 10 میلی گرم برکیلوگرم دریافت نمود. در ادامه داده ها جمع آوری شد و با استفاده از نرم افزار آماری17 SPSS و آنالیز واریانس یک طرفه در سطح معنی داری 05/0P< مورد تجزیه و تحلیل قرار گرفتند

Title: اثر دارچین بر شاخص های قندی و مقاومت به انسولین در موش های صحرایی نر بالغ دیابتی شده با استرپتوزوتوسین

http://yafte.lums.ac.ir/article-1-1795-fa.html

بحث و نتیجه‌گیری: دارچین احتمالاً با داشتن ترکیبات فلاونوئیدی و آنتی‌اکسیدانی از طریق افزایش برداشت گلوکز توسط سلول های مختلف بدن و با کاهش سطح استرس اکسیداتیو باعث اصلاح شاخص های قندی و انسولینی خون شده است یافته‌ها: نتایج نشان داد که در گروه‌های دریافت کننده عصاره دارچین شاخص‌های قندی و انسولینی به طور معناداری اصلاح گردید (05/0˂P). مواد و روش‌ها: این تحقیق یک مطالعه تجربی است که بر روی 40 سر موش صحرایی نر بالغ انجام شد. نمونه‌ها طور تصادفی به 4 گروه شامل کنترل غیردیابتی ،کنترل دیابتی و 2 دسته تجربی دریافت کننده دوز mg/kg60 عصاره دارچین به مدت 3 و 6 هفته تقسیم شدند. در پایان با خون‌گیری از قلب حیوانات میزان قند و انسولین، اندازه-گیری و با استفاده از اسکورHOMA مقاومت انسولینی مشخص گردید. نرمال بودن توزیع داده‌ها از طریق آزمون کالموگروف- اسمیرنوف و آنالیز نتایج با استفاده از نرم افزار SPSS-20 و با کمک آزمون‌های ANOVA و توکی انجام گرفت. در نهایت 05/0≥P از نظر آماری معنی دار در نظر گرفته شد. مقدمه: دیابت از بیماری‌های شایع می باشد که جهت درمان و کنترل آن، روش های درمانی مختلفی مانند استفاده از داروهای طبیعی و یا اصلاح شیوه زندگی به بیماران توصیه می شود. از آن جا که استفاده از داروهای گیاهی نسبت به بسیاری از داروهای شیمیایی از عوارض کمتری برخوردارند، این مطالعه با هدف بررسی اثر عصاره دارچین بر میزان قند خون، انسولین و مقاومت به انسولین در موش های صحرایی دیابتی شده با استرپتوزوتوسین انجام گردید

Title: شناسایی عوامل نوکاردیوزیس در نمونه های شستشوی برونش(BAL) بیماران مشکوک به سل مراجعه کننده به بیمارستان های تهران به روش PCR

http://yafte.lums.ac.ir/article-1-1796-fa.html

مقدمه: تشخیص گونه‌های نوکاردیای عامل نوکاردیوزیس در آزمایشگاه های روتین پزشکی بر پایه روش های فنوتیپی است که بسیار مشکل و اغلب طولانی و زمان بر است. هدف از این مطالعه شناسایی عوامل نوکاردیوزیس در نمونه های BAL بیماران مشکوک به سل مراجعه کننده به بیمارستان های تهران به روش PCR است. بحث و نتیجه‌گیری: در مطالعه حاضر استخراج DNA گونه های نوکاردیا در نمونه شستشوی برونش به روش دستی و در حداقل زمان انجام گرفت که سابقه قبلی در ایران نداشت. همچنین بررسی در افراد مشکوک به سل صورت گرفت که به صورت محدود کار شده است، گونه N. cyriacigeorgica گونه غالب عامل نوکاردیوزیس بود که طی سال های اخیر معرفی شده است و باید در آزمایشگاه ها، مراکز درمانی و تحقیقاتی مد نظر قرار گیرد. یافته‌ها: به روش مولکولی Duplex PCR ، نوکاردیوزیس در 7 نمونه (03/6%) تشخیص داده شد. پس از تعیین توالی محصولات PCR، مشخص شد که گونه‌های جداسازی شده متعلق به گونه‌های N. cyriacigeorgica (6 مورد) و N.otitidiscaviarum (1 مورد) می باشد. مواد و روش‌ها: در طی 8 ماه تعداد 116 نمونه شستشوی برونش (BAL) از بیماران بستری در بیمارستان های بقیه ا...(عج)، شریعتی و امام خمینی تهران جمع آوری گردید. استخراج DNA به روش فنل کلروفرم صورت گرفت. جهت انجام Duplex PCR از دو جفت پرایمر NG1 و NG2 اختصاصی جنس نوکاردیا و پرایمرهای BetF و BetR به عنوان ژن استفاده شد. به منظور شناسایی گونه عامل بیماری، محصولات PCR از روی ژل تخلیص و تعیین توالی گردید.

Title: تأثیر الگوی توانمندسازی خانواده محور بر کیفیت زندگی دختران نوجوان

http://yafte.lums.ac.ir/article-1-1797-fa.html

مقدمه: کم خونی فقرآهن شایعترین بیماری خونی درکودکان و نوجوانان می باشد و مانند سایر بیماری های مزمن کیفیت زندگی فرد مبتلا را تحت تأثیر قرار می دهد. مطالعه حاضر به منظور تعیین اثر الگوی توانمندسازی خانواده محور بر کیفیت زندگی دختران نوجوان مبتلا به کم خونی فقر آهن انجام گردیده است. بحث و نتیجه‌گیری: نتایج پژوهش نشان داد که اجرای الگوی توانمندسازی خانواده محور در افزایش کیفیت زندگی دختران نوجوان مبتلا به کم خونی فقر آهن مؤثر بوده است. یافته‌ها: قبل از مداخله میانگین امتیاز کیفیت زندگی نوجوان بین گروه آزمون و شاهد با آزمونt مستقل تفاوت معنی داری وجود نداشت در حالی که بعد از مداخله بین دو گروه با آزمون t مستقل اختلاف معنی داری از نظر میانگین نمرات کیفیت زندگی مشاهده گردید (000/0=P). همچنین با انجام آزمون t زوجی قبل و بعد از مداخله درگروه آزمون از نظر نمرات کیفیت زندگی تفاوت معنی داری مشاهده شد (000/0=P) در حالی که درگروه کنترل این اختلاف معنی دارنبود. مواد و روش‌ها: این پژوهش یک مطالعه نیمه تجربی از نوع کاربردی است که با انتخاب60 دخترنوجوان مبتلا به کم خونی فقرآهن در دو گروه آزمون و کنترل از طریق نمونه گیری تصادفی انجام شد. ابزارهای پژوهش شامل پرسشنامه های اطلاعات دموگرافیک وکیفیت زندگی اختصاصی نوجوانان (KIDSCREEN-52) بود. الگوی توانمندسازی خانواده محور در گروه آزمون اجرا و آزمون ثانویه 5/1 ماه بعد از مداخله انجام شد. درنهایت 05/0˂P معنی دار در نظر گرفته شد.

Title: مقایسه تأثیر دو داروی موضعی پیروکسیکام و املا بر شدت درد ناشی از کانولاسیون فیستول در بیماران همودیالیزی

http://yafte.lums.ac.ir/article-1-1798-fa.html

مقدمه: بیماران نارسایی مرحله نهایی کلیه، درد ناشی از کانولاسیون های مکرر فیستول را به عنوان شدیدترین استرس حاصل از روند درمان و بزرگ ترین دغدغه زندگی خود می دانند. پرستاران به عنوان یکی از اهداف اساسی اقدامات خود، وظیفه تسکین این درد را بر عهده دارند. براین اساس، این مطالعه با هدف تعیین تأثیر دو داروی موضعی پیروکسیکام و املا بر شدت درد ناشی از کانولاسیون فیستول در بیماران همودیالیزی انجام شد. بحث و نتیجه‌گیری: نتایج این مطالعه نشان داد که پماد املا در کاهش درد ناشی از کانولاسیون فیستول بیماران همودیالیزی از ژل پیروکسیکام مؤثر بوده است، لذا بر اساس نتایج این مطالعه می توان پیشنهاد نمود که از پماد املا به عنوان روشی آسان و با قابلیت کاربرد توسط خود بیمار، جهت کاهش درد حین کانولاسیون فیستول بیماران همودیالیزی استفاده شود. یافته‌ها: میانه کاهش شدت درد در گروه املا به وضوح بیشتر از دو گروه پیروکسیکام و پلاسبو بوده و این تفاوت معنی دار می‌باشد (001/0˂P) و در مورد مقایسه گروه پیروکسیکام و پلاسبو نیز مشخص شد که میانه کاهش شدت درد در گروه پیروکسیکام به مراتب بیشتر از گروه پلاسبو می باشد، ولی این مقدار در سطح معنی داری نبود. مواد و روش‌ها: این مطالعه به روش کار آزمایی بالینی بر روی 75 بیمار مراجعه کننده به بخش دیالیز بیمارستان شهدای عشایر خرم آباد 1392 انجام شد. بیماران به صورت تصادفی به سه گروه A (پیروکسیکام)، گروه B (املا) و گروه C (پلاسبو) تقسیم شدند. ابزار گردآوری اطلاعات پرسشنامه شامل سه بخش اطلاعات دموگرافیک، مقیاس دیداری اندازه گیری درد (VAS) و چک لیست عوارض جانبی احتمالی داروها بود. شدت درد حین کانولاسیون فیستول در سه گروه، در دو نوبت قبل و پس از مداخله اندازه گیری شد. به منظور تجزیه و تحلیل داده‌ها از آزمون‌های آماری کروسکال والیس و من ویتنی و نرم افزار SPSS19 استفاده شد.

Title: تعیین میزان تستوسترون سرمی در زنان سنین باروری مبتلا به مولتیپل اسکلروزیس و رابطه آن با شدت بیماری

http://yafte.lums.ac.ir/article-1-1799-fa.html

مقدمه: بیماری مولتیپل اسکلروزیس (MS) مهمترین بیماری تخریب غشاء میلین می باشد. برای علت آن یک پایه خودایمنی شناخته شده و نیز مطالعات نیز نشان داده است که تغییرات هورمونهای جنسی از جمله تستوسترون نقشی در این مکانیسم دارد. هدف این مطالعه تعیین این تغییرات و رابطه آن با شدت بیماری می باشد. بحث و نتیجه‌گیری: تستوسترون ارتباط مثبتی با بیماری ام اس دارد. بنابراین مطالعات دیگری در جهت بررسی ارتباط و تآثیر سیستم آندوکرین در علت و درمان بیماری ام اس باید انجام گیرد. یافته‌ها: در این مطالعه سطح سرمی تستوسترون در بیماران در طی مرحله فولیکولی 3/0±0461/0 و گرم در هر میلی لیتر بود در مقایسه گروه کنترل 0.40/0 ±0.82 (011/0P = ) و در طی مرحله لوتئال 7/0 ±58/0 نانوگرم در هر میلی لیتر بود در مقایسه گروه کنترل 3/0±88/0 (03/0P = ) مشاهده شد. بنابراین میانگین میزان سرمی تستوسترون از نظر آماری تنها در هر دو مرحله لوتئال و فولیکولی سیکل قاعدگی به طور معنی داری پایین می باشد (05/ 0P <)، ولی ارتباطی با شدت بیماری ملاحظه نشد. مواد و روش‌ها: 32 بیمار مبتلا به مولتیپل اسکلروزیس در این مطالعه مقطعی انتخاب شدند و با تعداد 60 نفر سالم بعنوان گروه کنترل همسان سازی شدند و میزان سرمی هورمون تستوسترون در هر دو گروه به طور تصادفی در دو مرحله لوتئال و فولیکولی سیکل قاعدگی تعیین و سپس رابطه میزان هورمون با شدت بیماری بررسی گردید.
